# Cancer treatment–related bone loss: a review and synthesis of the literature

**DOI:** 10.3747/co.2008.174

**Published:** 2008-01

**Authors:** M.N. Khan, A.A. Khan

**Keywords:** Cancer therapy, aromatase inhibition, bone loss, osteoporosis, androgen deprivation, antiresorptive therapy, bisphosphonates, osteonecrosis of jaw

## Abstract

Cancer therapy can result in significant bone loss and increased risk of fragility fracture. Chemotherapy, aromatase inhibitors, and gonadotropin-releasing hormone analogues contribute to increases in the rate of bone remodelling and reduce bone mineral density. Patients with prostate cancer on androgen deprivation therapy experience an increase in the risk of fracture. New research has demonstrated the key role played by bisphosphonates in preventing declines in bone density and increases in bone remodelling. Novel antiresorptive agents targeting receptor activator of nuclear factor κB ligand have great potential in skeletal protection and prevention of bone loss related to cancer therapy. Early assessment of skeletal health, followed by initiation of calcium, vitamin D, and an exercise program are valuable in the prevention and treatment of osteoporosis. In addition, individuals at increased risk for fracture should be offered antiresorptive therapy. Early data have demonstrated that bisphosphonates are able to prevent the bone loss and increased bone remodelling associated with cancer therapy, including aromatase inhibition and androgen deprivation therapy. The present paper reviews the new research and advances in the management of bone loss associated with both cancer therapy and estrogen deficiency in the postmenopausal female.

## 1. INTRODUCTION

Osteoporosis is characterized by a decline in bone mineral density (bmd) and an associated deterioration of bone microarchitecture, resulting in increased bone fragility and higher fracture risk 1. Osteoporotic fractures result in increased morbidity and mortality, with men experiencing higher mortality rates following a hip fracture (close to 40% within the first year of the fracture). Oncology patients are at an increased risk of developing osteoporosis because of the effects of chemotherapy and radiotherapy. Hormonal and non-hormonal treatment options can both also result in hypogonadism, which can further contribute to progressive bone loss.

## 2. DISCUSSION

### 2.1 Aromatase Inhibitors, Gonadotropin-Releasing Hormone Analogues, and Tamoxifen

In premenopausal women with breast cancer, ovarian ablation therapy can be associated with a rate of bone loss as high as 13% within 12 months of treatment 2. Gonadotropin-releasing hormone (gnrh) analogues result in downregulation of gnrh receptors and ovarian insufficiency within 6 months of therapy 3. Dramatic decreases in bone density have been observed in patients treated with goserelin as compared with patients treated with cyclophosphamide, methotrexate, and 5-fluorouracil (cmf) adjuvant chemotherapy 4. In goserelin-treated patients, bmd at the lumbar spine decreased by 10.5%, as compared with 6.5% in cmf-treated patients (*p* < 0.001) 4. The cmf patients remained estrogen-deficient and had ongoing bone loss, whereas patients in the group treated with goserelin demonstrated bone density recovery 1 year after therapy ended 5.

The aromatase inhibitors (ais) block conversion of androgens to estrogen and can also contribute to bone loss 6. As a steroidal ai, exemestane has a structure similar to androstenedione and competitively binds with aromatase. The nonsteroidal ais anastrozole and letrozole reversibly inhibit the cytochrome P450 moiety of aromatase. All three of these agents have been approved for adjuvant treatment of breast cancer. The major metabolite of exemestane, 17-hydroexemestane, has androgenic effects, and the steroidal and nonsteroidal ais may differ with respect to their effects on bone loss and fracture. These potential differences have not been evident in clinical trials completed to date.

All three ais have been associated with increases in markers of bone resorption and bone formation, and no statistically significant differences have been noted between the three agents 7–9. All three appear to increase the rate of bone turnover and thus may be associated with an increased risk of fracture 7–9. The clinical trials completed to date have been small in size, and the effect of ais on fracture risk is not yet clear, partly because in the trials completed to date, the ais have been compared to tamoxifen. Tamoxifen exerts estrogen agonistic effects at the skeletal level and can reduce the rate of bone remodelling and provide skeletal protection. Thus it is not the ideal standard against which to measure the skeletal effects of ais.

In postmenopausal women, tamoxifen has been shown to preserve bone density and to reduce the rate of fracture 10–12. In premenopausal women, tamoxifen is associated with bone loss at the spine and hip because its effects on the skeleton are not as potent as those of estrogen. Tamoxifen is thus acting as a bone antagonist as it competes with the more potent bone agonist 17β-estradiol for the estrogen receptor in the premenopausal female. Small reductions in bone density at the lumbar spine were noted over 3 years in premenopausal women treated with tamoxifen as compared with women receiving placebo 11. In postmenopausal women, tamoxifen was associated with increases in bone density of 1.17% at the lumbar spine and 1.71% at the hip (*p* < 0.001); the placebo group showed stable bone density 11. In a primary prevention breast cancer study 13, 5 years of tamoxifen therapy were associated with a 32% reduction in osteoporotic fracture [95% confidence interval (ci) = 0.51 to 0.92].

The Arimidex, Tamoxifen, Alone or in Combination (atac) and the Breast International Group (big) trials 14,15 confirmed that adjuvant therapy with anastrozole or letrozole was superior to tamoxifen therapy with respect to breast cancer outcome. Eastell and colleagues 8 concluded that anastrozole is associated with significant bmd loss and a small increase in bone turnover, but that tamoxifen or a combination of anastrozole and tamoxifen is associated with increased bmd and decreased remodelling. For anastrozole, significant loss of bmd at the lumbar spine and hip was noted at both year 1 and year 2; with tamoxifen, significant gains occurred at the lumbar spine and the hip at year 1 and year 2 8. An inverse correlation was seen between the baseline estradiol level and bmd change: a lower baseline estradiol level was predictive of greater bmd losses.

The atac trial included 9366 patients treated for 5 years. In that study, the bone resorption marker urinary N-telopeptide of type 1 collagen (ntx) was measured, as was the resorption marker serum C-telopeptide of type 1 collagen (ctx) and the formation markers procollagen type 1 N-propeptide (p1np) and serum bone-specific alkaline phosphatase (bone alp). Patients receiving anastrozole showed increases in bone turnover markers; patients receiving tamoxifen tended to show decreases. At 1 year, the resorption markers ctx and ntx increased by 26% and 15% respectively (95% ci: 3% to 25%) in the anastrozole arm. The formation markers p1np and bone alp increased by 18% and 20% respectively (95% ci: 14% to 25%). Tamoxifen, however, was associated with decreases in bone resorption: ctx decreased by 56% and ntx, by 52% (95% ci: –62% to –33%); p1np decreased by 72% and bone alp, by 16% (95% ci: –24% to –11%). The combination of anastrozole plus tamoxifen was comparable to tamoxifen alone with respect to changes in bone markers. Anastrozole treatment for 2 years was associated with bone loss at the hip and spine and with increases in bone turnover 8.

The atac study demonstrated that the low levels of estrogen present in postmenopausal women are important in preserving bone density and regulating bone turnover. The effects of anastrozole were most notable within the first 4 years after menopause, a period associated with a rapid rate of bone loss. Tamoxifen was associated with skeletal protection and resulted in preservation of bone density and a decrease in bone turnover. However, tamoxifen in combination with anastrozole was less effective in improving disease-free survival than was anastrozole alone. The combination is therefore not recommended in the management of postmenopausal women with breast cancer.

The atac trial found that anastrozole had a safer profile than tamoxifen with respect to endometrial cancer, cerebrovascular events, and venous thromboembolic events 14. However, as compared with tamoxifen, anastrozole was associated with an increase in the overall fracture rate (11% vs. 7.7%, *p* < 0.0001)^16^. In the big 1–98 trial, a higher incidence of clinical fractures was noted in the letrozole group as compared with the tamoxifen group (5.7% vs. 4%, *p* < 0.001) 15. However, these trials make it difficult to confirm the effects of ais on fracture, because the control group was treated with tamoxifen, which has protective skeletal effects and preserves bone density.

The effect of tamoxifen on fracture has not yet been confirmed. How an agent affects vertebral fracture is best evaluated by serial X-ray of the spine. Unfortunately, the large trials conducted to date have not included serial spinal radiographs as part of the clinical assessment, and therefore the effect of ais on vertebral fracture is difficult to define.

The use of ais after tamoxifen therapy has been evaluated. Exemestane was studied in 4742 postmenopausal women with breast cancer who had previously received 2–3 years of tamoxifen therapy 17. Patients were randomized to switch to exemestane for the remainder of the 5-year treatment program or to complete a full 5 years on tamoxifen. After 30 months of follow-up, a higher, but not statistically significant, clinical fracture rate was noted in the exemestane group as compared with the tamoxifen group (3.1% vs. 2.3%, *p* = 0.08). A new diagnosis of osteoporosis was more common in the exemestane group (7.4% vs. 5.7%, *p* = 0.05) 17.

Anastrozole after tamoxifen has also been associated with a greater number of clinical fractures than has tamoxifen alone (2% vs. 1%, *p* < 0.05) 18. But the effect of the ai on fracture is difficult to interpret, because tamoxifen, being an estrogen agonist in the postmenopausal female, may have reduced the risk of fracture. The study included premenopausal and postmenopausal women alike, and did not obtain spinal radiographs. These limitations make it difficult to confirm the effect of the ai on the incidence of fracture.

In a 2-year randomized control trial 19, exemestane was compared to placebo in postmenopausal women with early breast cancer. Assessments of spine and hip by dual X-ray absorptiometry were completed on an annual basis. After 1 year, the exemestane group showed declines in bone density at the lumbar spine (2.2%) and the hip (2.7%). The placebo group also experienced declines in bone density (1.8% at the spine, 1.5% at the hip). There was no significant difference between the two groups at the spine; the difference at the hip was statistically significant at *p* = 0.023 19. Exemestane-treated patients demonstrated increases in bone remodelling, with an increase of 44% in p1np and 35% in ctx. The increased rates of bone turnover were consistent with the data obtained from other clinical trials.

Eastell and colleagues evaluated risk factors for fracture in the atac study^8^. Older age was associated with a higher risk of fracture. Low bone density T scores of less than –2.5 were also associated with a higher risk of fracture than were bone density T scores greater than –1.5. These authors also noted that use of a statin was associated with a lower risk of fracture.

Androgen deprivation therapy has been evaluated with respect to its effects on bone loss and fracture. Gonadotropin-releasing hormone agonists reduce serum concentrations of testosterone by more than 95% 20. That decline is associated with dramatic reductions in bone density of about 4%–10% within the first year of treatment 21. An increased risk of fragility fractures was documented retrospectively with androgen deprivation therapy in men with prostate cancer 22–24. In a retrospective review of 50,613 men who received a diagnosis of prostate cancer, 19.4% of those who received androgen deprivation therapy had a fracture, as compared with 12.6% of those who did not receive such therapy (*p* < 0.001) 16. Duration of treatment was an independent predictor for fracture risk. Men who received 9 or more doses of gnrh agonists in the year after diagnosis and men who underwent orchiectomy both had a relative risk of fracture of 1.45 (95% ci: 1.36 to 1.56)^16^.

Bicalutamide, an approved agent for prostate cancer, is a nonsteroidal anti-androgen. It competitively binds to the androgen receptors in target tissues, inhibiting the effects of androgen. It can increase serum concentrations of estradiol 25. As compared with gnrh analogues, bicalutamide has been shown to increase bmd^26^.

### 2.2 Prevention and Treatment of Osteoporosis

In addition to adequate calcium, vitamin D, and exercise, options for the prevention and treatment of osteoporosis include antiresorptive and anabolic agents.

Antiresorptive (anticatabolic) agents inhibit osteoclast activity and reduce bone turnover 2, with the various agents having different mechanisms of action. Bisphosphonates reduce the rate of bone turnover, providing a longer time for bone to mineralize. Bisphosphonate therapy is thus associated with modest increases in bmd. Estrogen acts through the estrogen receptors on both osteoblasts and osteoclasts, and results in suppression of receptor activator of nuclear factor κB ligand (rankl)–induced osteoclast differentiation, thereby decreasing bone remodeling 3. Raloxifene, a selective estrogen receptor modulator (serm), can bind to estrogen receptors, with tissue-specific agonist or antagonist effects. Raloxifene decreases bone remodelling, in addition to its other extraskeletal effects. Osteoclastic bone resorption is also inhibited by calcitonin acting on calcitonin receptors.

The antiresorptive agents mentioned above are effective in reducing fracture risk by approximately 30%–50% in the postmenopausal female population. However, fractures still occur, and more effective options are desirable, particularly for severe disease states. Anabolic therapy can make major improvements in the quality and quantity of bone, and anabolic agents are a welcome addition to the antiresorptive options currently available.

Anabolic therapy can increase the production of bone matrix by enhancing osteoblastic function. The resulting reduction in risk of fracture is approximately 65% over 18 months in the postmenopausal female with osteoporosis 27. The currently approved anabolic agent is teriparatide. Strontium ranelate has been shown to have antiresorptive properties; it may also have anabolic properties. This agent is expected to be approved in Canada for the treatment of postmenopausal osteoporosis before the end of 2008.

#### 2.2.1 Calcium and Vitamin D

The Scientific Advisory Council of Osteoporosis Society of Canada recommends 28

- 1500 mg elemental calcium daily and 800 IU vitamin D daily for women and men 50 years of age and older.
- 400 IU vitamin D daily and 1000 mg elemental calcium daily for all adults under 50 years of age.

The effectiveness of calcium and vitamin D in preventing hip fracture was evaluated in the Women’s Health Initiative (whi) trial, which involved 36,282 postmenopausal women who daily received either 1000 mg calcium and 400 IU vitamin D, or a placebo, for an average of 7 years 29. Patients were allowed to take additional daily supplements of up to 1000 mg calcium and 600 IU vitamin D. Personal use of bisphosphonates, calcitonin, serms, and estrogen therapy was also permitted, and approximately 38% of subjects took more than 1200 mg elemental calcium daily. In the whi, the calcium and vitamin D study arm overlapped with the hormone replacement therapy (hrt) arm, and thus approximately 51% of patients were receiving estrogen.

Treatment compliance was poor: by the end of the study, only 59% of patients were taking more than 80% of their medication. As compared with the patients taking placebo, the patients taking 1000 mg calcium and 400 IU vitamin D daily showed a 1.06% increase in hip bmd (*p* < 0.01). In the treatment-compliant group, the hazard ratio for hip fracture was 0.71 (95% ci: 0.52 to 0.97), representing a statistically significant 29% reduction in hip fracture risk in the individuals taking more than 80% of their calcium and vitamin D supplements. Estrogen use was associated with a 42% reduction in hip fracture risk. A small, but significant, 17% increase in risk of renal stones was noted in the treatment group as compared with the placebo group, with a hazard ratio of 1.17 (95% ci: 1.02 to 1.34) 29. Clinicians must therefore ensure that patients are not inadvertently taking excessive calcium supplements and that their urinary calcium excretion is normal, particularly when a history of renal stone formation is present.

Vitamin D inadequacy was also noted in the whi study and may have contributed to the findings. In the nested case–control study, the mean 25-hydroxy vitamin D level was 46 nmol/L in patients who sustained hip fractures as compared with 48.4 nmol/L in control subjects 29. Vitamin D supplementation of more than 600 IU daily may have reduced the fracture risk, as has been demonstrated in other clinical trials. Ensuring adequate vitamin D supplementation is a key component of therapy in the prevention and treatment of osteoporosis.

#### 2.2.2 Antiresorptive Therapy

##### SERMS

The serm class of agents demonstrates tissue-specific estrogen-agonistic or estrogen-antagonistic effects 30. In the Multiple Outcomes of Raloxifene Evaluation trial, patients treated with either 60 mg or 120 mg raloxifene daily for 4 years demonstrated a 36% and 43% reduction in vertebral fracture risk respectively 31. However, no significant effect on the risk of non-vertebral fractures was noted 31. The latter finding may have been the result of multiple factors, including the very low incidence of non-vertebral fractures seen in the placebo arm of this trial, as compared with incidences seen in the randomized clinical trials for other antiresorptives.

In the Study of Tamoxifen and Raloxifene, involving 19,747 postmenopausal women with increased risk of breast cancer, 60 mg raloxifene daily demonstrated breast cancer risk reduction effects equivalent to 20 mg tamoxifen daily over 5 years 32. (Both drugs reduce the risk of breast cancer by approximately 50%.) Raloxifene was associated with a better overall safety profile than tamoxifen was, with 36% fewer uterine cancers and 29% fewer deep vein thromboses 32.

##### Bisphosphonates

Nitrogen-containing bisphosphonates (alendronate, risedronate, zoledronic acid) demonstrate antiresorptive effects by binding to the calcium hydroxylapatite crystal at sites of bone resorption where the bone matrix is exposed 33. The bisphosphonate is buried under the newly formed bone, where it lies inert and has no skeletal effects. During bone resorption, the drug is released from the bone matrix and is ingested by osteoclasts; it inhibits farnesyl diphosphate synthase (fdps), a key enzyme in the cholesterol synthesis pathway involved in post-translational modification of important signalling molecules (Ras, Rac, Rho, and Rab). The fdps inhibition disrupts several pathways involved in cytoskeletal organization, cell survival, and cell proliferation, leading to osteoclast deactivation and apoptosis 34. The result is reduced bone turnover and enhanced bone mineralization because of the extended time available for mineral accumulation. With the normalization of bone remodelling to premenopausal levels, overall bone strength is improved 34,35.

###### Alendronate

Alendronate effectively reduces the risk of vertebral fractures in postmenopausal women with and without baseline vertebral fractures, as has been demonstrated in the Fracture Intervention Trial (fit)^36^–^38^. Several trials have shown that alendronate use reduces bone resorption and improves bmd 38,39. In early postmenopausal women, alendronate for 5 years inhibited bone loss at the spine, hip, and total body 37. In a separate study involving women below 60 years of age, 5 mg alendronate daily resulted in a 3.5% increase in bmd at the lumbar spine, together with a 1.9% increase at the hip (*p* < 0.001 as compared with baseline at both sites) 39. It is worth noting that the fit Long-term Extension study reported that increases in bmd continued at the lumbar spine and hip through 10 years of treatment, with an associated fracture risk reduction 38. Bone biopsies performed in patients following 10 years of alendronate treatment revealed double fluorescent tetracycline label in all biopsy samples, indicating ongoing bone remodelling and an absence of “frozen” bone 38. Those data provides reassurance that long-term alendronate therapy can safely reduce vertebral and non-vertebral fractures.

Bisphosphonates may prevent bone loss associated with gnrh-induced hypogonadism, as was first evaluated with etidronate 40. Clinical trials are currently evaluating the use of alendronate or risedronate in the prevention of bone loss in association with ais.

###### Risedronate

Risedronate maintains bone mass and preserves bone microarchitecture 41. A number of studies in postmenopausal women have shown that risedronate significantly reduces the risk of vertebral and non-vertebral fractures alike 42–44. Analysis of data from the Vertebral Efficacy with Risedronate Therapy trials indicated that 5 mg risedronate daily reduced the incidence of new fractures within 6 months of starting therapy and significantly lowered the risk of new vertebral fractures within 1 year 42–44. This reduction in fracture risk was maintained for up to 7 years of treatment 45. Risedronate effectively reduced the risk of hip fractures in a study evaluating 9331 elderly women at high risk of fracture 41.

Risedronate has been shown to effectively reduce the risk of non-vertebral fractures after 3 years of treatment 46. Another study, involving early postmenopausal women, demonstrated that, as compared with placebo, 5 mg risedronate daily increased bmd at the lumbar spine by more than 5% during 2 years of treatment (*p* < 0.05 as compared with both baseline and placebo) 47. Other studies have confirmed those findings, showing that risedronate prevents bone loss and preserves trabecular architecture in early postmenopausal women 48. In addition, key clinical trials have shown that reductions in vertebral fracture risk with risedronate are independent of increases in bmd 41.

The Study of Anastrozole with the Bisphosphonate Risedronate evaluated the effects of risedronate on bmd and bone turnover in postmenopausal women using anastrozole as adjuvant therapy for hormone receptor–positive early breast cancer 49. The pre-planned analysis of markers of bone turnover after 6 months of therapy were presented in June 2007 49. Patients were stratified according to baseline fracture risk by lumbar spine and hip T scores, with higher risk defined as including patients with a T score of less than –2 or a history of fracture, or both. “Moderate risk” included patients with a T score of less than –1, but greater than or equal to –2. “Low risk” included patients with a T score of –1 or better. High-risk patients received open-label anastrozole plus risedronate 35 mg once weekly. Moderate-risk patients were randomized in a double-blind manner to receive anastrozole plus risedronate or placebo. Low-risk patients received open-label anastrozole only. All patients were given calcium and vitamin D. Low-risk patients demonstrated a significant increase in serum ctx (*p* = 0.05), but no significant change in p1np or bone alp. Significant decreases in all bone markers were noted for the high-risk patients (*p* < 0.0001). In the moderate-risk group, all markers significantly declined in patients receiving anastrozole plus risedronate as compared with patients receiving anastrozole plus placebo (*p* < 0.0001). Risedronate was able to reduce bone turnover in anastrozole-treated postmenopausal women with hormone receptor–positive early breast cancer and a pre-existing moderate or higher risk of fracture 49.

###### Zoledronic Acid

Zoledronic acid is the most potent bisphosphonate currently available 50,51. It contains two nitrogen atoms in the R2 side chain, and the intravenous administration of 4-mg doses has been approved for the prevention and treatment of metastatic bone disease and hypercalcemia of malignancy. Zoledronic acid 5 mg has been approved in Canada as a treatment for Paget disease.

In a double-blind placebo-controlled dose-ranging trial involving 351 postmenopausal women with low bmd, significant reductions in markers of bone resorption were noted, together with improvements in bmd, in all treatment groups over placebo^52^. In addition, the study noted that zoledronic acid 4 mg annually achieved reductions in bone turnover comparable to those seen with daily oral bisphosphonate therapy 52.

The phase iii randomized controlled Health Outcomes and Reduced Incidence with Zoledronic Acid Once Yearly (horizon) pivotal fracture trial (pft) evaluated the effects of zoledronic acid 5 mg annually on fracture incidence in men and women 50 years of age or older who had already sustained a low-trauma hip fracture 53. Data from that trial demonstrated that zoledronic acid reduced the 3-year risk of vertebral fractures by 70% over placebo (relative risk: 0.30; 95% ci: 0.24 to 0.38). The 3-year fracture risk for hip fractures was reduced by 41% in the zoledronic acid group over placebo. Increases in bmd were significantly higher and height loss was lower in the zoledronic acid group 53.

In another study, 301 patients receiving adjuvant letrozole were randomized to upfront or delayed zoledronic acid given intravenously in doses of 4 mg every 6 months 54. Zoledronic acid was administered when the lumbar spine or total hip T score dropped below –2 or in the presence of a non-traumatic fracture. At 12 months, lumbar spine bmd was 4.4% higher in the upfront group than in the delayed start group. Bone resorption marker was also 15% lower in the upfront group. Zoledronic acid has been approved for the prevention of osteoporosis in the postmenopausal female population. Further evaluation of this valuable agent in preventing bone loss induced by cancer treatment is planned.

###### Bisphosphonate Advantages and Disadvantages

A major advantage of oral bisphosphonate therapy is ease of administration and an excellent tolerability profile. The side effects most commonly associated with such therapy are abdominal pain and dysphagia. However, alendronate and risedronate have both demonstrated upper gastrointestinal side effects comparable to placebo in the randomized controlled trials conducted to date 55. Intravenous administration of bisphosphonates provides a number of advantages, including less frequent dosing and less potential for gastrointestinal side effects as compared with oral bisphosphonates.

Recently, reports of mandibular or maxillary osteonecrosis as a potential rare complication of bisphosphonate use have been published 56. Osteonecrosis of the jaw (onj) is an avascular bone necrosis that may occur in patients at risk for this condition. Most of the reports have been associated with frequent high-dose administration of intravenous pamidronate or zoledronic acid in oncology patients with a history of breast cancer or myeloma. Many of these patients have been on concomitant chemotherapy or radiotherapy (or both), which are risk factors for avascular bone necrosis. The condition has been most commonly reported in high-risk individuals following dental surgery such as dental extraction 57. The condition has seldom been reported with alendronate and risedronate use 56. It is important to note that onj has not been seen in any of the clinical trials conducted to date, which represent prospective data obtained in more than 100,000 patients treated with aminobisphosphonates for an average of 3 years. All published cases have been anecdotal reports. In a retrospective chart review by the M.D. Anderson Cancer Center of 4000 cancer patients treated with zoledronic acid, pamidronate, or both, onj was identified in 0.825% of the patient population 58. The horizon pft trial found that the incidence of onj was similar in the treatment and placebo groups, with 1 case occurring in each group. The cases were validated by an adjudication committee.

Canadian guidelines regarding the diagnosis, prevention, and management of onj are expected to be published in early 2008 by a Canadian multidisciplinary task force on onj. Current international guidelines recognize onj as a very rare condition limited mostly to the oncology population receiving high-dose intravenous bisphosphonates. Prospective data in the oncology and non-oncology population are needed to better understand the underlying pathophysiology of onj so that appropriate decisions can be made about prevention, diagnosis, and management.

Administration of potent bisphosphonates has been shown to provide a number of advantages in patients with metastatic disease, such as the ability to effectively control hypercalcemia and to stabilize bone lesions while bypassing the gastrointestinal tract. Thus, intravenous bisphosphonates are of tremendous value in the management of skeletal complications in patients with malignancy, and they are an important component of therapy in those patients. The benefits of intravenous bisphosphonates far outweigh the risks of developing onj. Further prospective data are needed for the true incidence and pathophysiology of onj to be understood. The exact relationship between onj and the use of bisphosphonates also needs to be clarified. It should also be noted that the annual dose of intravenous bisphosphonates used in osteoporosis is significantly lower than that used in cancer patients.

Bisphosphonates are a valuable treatment option for postmenopausal osteoporosis as well as for steroid-induced bone loss. These agents may also be effective in preventing bone loss associated with cancer therapy; however, they have not yet been approved specifically for that indication.

##### HRT

Hypogonadism may be the result of cancer therapy in non-hormone dependent tumours such as lymphoma. In those circumstances, hypogonadism will be associated with progressive bone loss and is not an intended effect of cancer treatment. Data regarding estrogen replacement in the postmenopausal female has confirmed significant improvements in bmd, reductions in the rate of bone turnover, and reductions in the risk of vertebral and non-vertebral fracture.

Estrogen therapy has significant antiresorptive effects. Specifically, it enhances the osteoblastic production of osteoprotegerin, which has anti-osteoclastic properties because of its ability to bind to rankl and subsequently to block the rankl/rank interaction required for osteoclast recruitment and activation 59,60. In the whi, a primary prevention trial, the combined estrogen plus progesterone arm demonstrated an increase in total hip bmd, together with a 34% reduction in hip and vertebral fractures and a 24% reduction in total osteoporotic fractures 61. The estrogen-only arm presented similar results, with a 30%–39% reduction in fracture rates 62. This trial therefore confirmed the antifracture effects of hrt suggested by previous clinical trials 63,64. In early postmenopausal women, combined estrogen–progesterone therapy resulted in increases in bmd of 2%–3% at the hip and spine over 2 years of therapy 63. A decline in the markers of bone turnover in response to hrt was also seen in early postmenopausal women 64. The combined estrogen and progesterone arm experienced a 26% increased risk of breast cancer and a 29% increased risk of cardiac events, prompting the early termination of this treatment group after 5.2 years of follow-up 65. The estrogen-alone arm was also terminated prematurely because of a 41% increased risk of stroke and a doubling of the risk of thromboembolic disease 62. Because the risks of hrt are significant and appear to outweigh the benefits, hrt is considered a treatment option for select patients unsuited to standard first-line antiresorptive therapies 66.

##### RANKL Inhibitors

Denosumab is a fully human monoclonal antibody to rankl; it binds to human rankl, thus preventing osteoclast activation and subsequently reducing bone resorption. A phase ii study reported by McClung *et al.* demonstrated that denosumab was well-tolerated and had an effect on bone turnover similar to that of alendronate 67. As compared with patients who received placebo, patients treated with denosumab experienced significantly increased bmd. Subcutaneous administration of the drug in doses of either 30 mg every 3 months, or 60 mg every 6 months, resulted in sustained decreases in urinary N-telopeptide excretion 67.

In addition, denosumab has been shown to effectively reduce bone turnover in patients with metastatic bone disease, with effects comparable to those of intravenous pamidronate 68. This agent is currently being evaluated further in oncology patients with metastatic skeletal disease and in patients with postmenopausal osteoporosis. It is expected to become a valuable additional option in the management of those conditions.

#### 2.2.3 Anabolic Agents

Until recently, postmenopausal osteoporosis treatment was limited to antiresorptive therapies. However, the availability of anabolic agents—namely, parathyroid hormone (pth) and strontium ranelate—represent a major advance. Anabolic agents result in major improvements in the quality and the quantity of bone, significantly increasing bone strength.

##### PTH—Teriparatide and Full-Length PTH

Teriparatide, a 34-amino acid recombinant fragment of the 84–amino acid human pth, is administered subcutaneously in doses of 20 μg daily for 18 months. Although exposure to continuously elevated pth levels is associated with bone loss, intermittent pulse therapy with pth preferentially stimulates osteoblast activity and is associated with increases in bmd 69. Teriparatide promotes the differentiation of pre-osteoblasts into bone-forming osteoblasts 70, resulting in a net increase in both the number and activity of bone-forming cells 69. Several clinical trials have noted impressive effects of teriparatide on bmd and fracture risk 27,71–75.

In a randomized controlled trial involving post-menopausal women with fragility fractures, teriparatide 20 μg daily over 21 months led to a 9% increase in lumbar spine bmd and improved bmd at the femoral neck and whole body 27. Risks for vertebral and non-vertebral fractures were reduced by 65% and 53% respectively 27. Evidence for the anabolic effects of teriparatide on bone microarchitecture has been found in bone biopsies of patients treated with teriparatide. These show dramatic increases in the thickness, density, and number of trabeculae and increases in cortical thickness and bone size 71. A reduction in back pain has also been noted with teriparatide use.

The full-length pth molecule (1–84) has been assessed in the Treatment of Osteoporosis trial 72. That 2-year study involved 2532 postmenopausal osteoporotic women who were randomized to receive 100 μg pth (1–84) or placebo. The pth was found to reduce the incidence of new vertebral fractures by 66%.

Teriparatide is well-tolerated; however, minor adverse events such as nausea, headaches, and transient mild hypercalcemia have been noted 70. Preliminary studies in rats, in which the animals received near-lifelong exposure to high doses of teriparatide (5 μg/kg or more daily), found a dose- and duration-dependent relationship between teriparatide and the development of osteosarcoma 76. However, such doses are much higher than the 20 μg daily dose (approximately 0.28 μg/kg daily) used in humans, and osteosarcoma has not been seen in humans or in studies with monkeys. Furthermore, it is important to note that osteosarcoma does not occur with increased frequency in individuals with primary hyperparathyroidism. To date, 300,000 people have been treated with teriparatide, and 1 case of osteosarcoma has occurred, which is comparable to the background incidence of osteosarcoma of 1 in 250,000.

Teriparatide is contraindicated in individuals who have had metastatic skeletal disease or who have a history of skeletal radiation. It is also contraindicated in patients with renal insufficiency, hypercalcemia, hypercalciuria, or elevated alp that has not yet been evaluated. Because osteosarcoma is more common in individuals with Paget disease, teriparatide is contraindicated in those patients.

Combination therapies have included teriparatide in combination with hrt or with bisphosphonates 73,74. Data suggest that pretreatment with alendronate may blunt the anabolic effects of pth therapy 77. Long-term fracture risk studies are required to determine if combination therapy is of value.

##### Strontium Ranelate

Strontium ranelate has demonstrated antiresorptive effects and also appears to have anabolic properties. It has not been associated with an increased risk of osteosarcoma. It is incorporated into bone and accumulates in the skeleton because of its physical and chemical similarities to calcium 78.

Strontium ranelate provides skeletal benefits because it stimulates replication of pre-osteoblasts and synthesis of bone matrix; it also prevents bone resorption by inhibiting osteoclasts 79–83. In a study involving ovariectomized rats, strontium ranelate prevented bone loss by reducing bone resorption and enhancing bone formation 84. A phase iii trial involving 1649 postmenopausal women demonstrated a 49% reduction in new vertebral fractures at 1 year in subjects administered strontium ranelate 2 g daily as compared with those receiving placebo 85. A 16% reduction in non-vertebral fractures in the strontium ranelate group was also noted 86. Subjects at high risk of fracture (74 years of age or older with a femoral neck bmd T score of –3 or lower) demonstrated an even greater reduction in risk of hip fracture (36%, *p* = 0.046) 58. Side effects associated with strontium ranelate have been limited to nausea and diarrhea during the first few months of therapy 80. Current guidelines recommend that intervention be based on the presence of low bmd and planned cancer treatment.

### 2.3 Available Guidelines

International guidelines have been published providing recommendations for skeletal protection in oncology patients, including guidelines from the American Society for Clinical Oncology. A 2007 consensus statement published by the Belgian Bone Club provides recommendations for the management of bone loss induced by cancer treatment in early breast and prostate cancer 87. Their recommendations include assessment of fracture risk and bone density testing by dual X-ray absorptiometry for all women beginning medical castration therapy or aromatase therapy. All men beginning androgen deprivation therapy should similarly be evaluated for risk of osteoporosis. Monitoring of bmd every 1–2 years for osteoporotic and osteopenic patients is recommended. In individuals with a normal baseline bmd, that assessment is recommended be repeated every 2–5 years, depending on the presence of other risk factors. Lifestyle modification with appropriate calcium and vitamin D supplementation is also recommended. Further evaluation to exclude secondary causes of osteoporosis such as vitamin D deficiency, hyperparathyroidism, or hyperthyroidism is recommended to ensure that no other factors are contributing to bone loss apart from androgen deprivation therapy or induction of hypogonadism.

Bisphosphonate therapy is recommended for osteoporotic individuals with a T score of –2.5 or lower, or a history of a fragility fracture. Bisphosphonates are also recommended for individuals who have important risk factors for fracture and osteopenia with a T score between –1 and –2.5. In the latter case, the recommendations suggest zoledronic acid 4 mg every 6 months for patients receiving ais and zoledronic acid 4 mg annually or alendronate weekly for patients receiving androgen deprivation therapy.

Ongoing clinical trials evaluating the skeletal protective effects of bisphosphonates will provide further refinement of the existing recommendations.

## 3. SUMMARY

Osteoporotic fractures result in significant morbidity and increased mortality rates. Cancer treatment can dramatically reduce bone density and increase the rate of bone turnover and the risk of fragility fracture. The importance of both quantity and quality of bone in determining bone strength and fracture risk has been recognized. Bisphosphonates are being evaluated for their skeletal protective effects, and they offer great promise in the prevention of fragility fracture for patients receiving cancer treatment. Clinical research is also evaluating rankl inhibitors, and other antiresorptive options and anabolic therapies are expected to possibly play a future role in the prevention of fracture in the patient with cancer. Early diagnosis and treatment of osteoporosis will contribute to improved health and wellbeing for the patient receiving cancer treatment.

## 4. DISCLOSURE

Aliya Khan has received research funding from Lilly, Merck, Novartis, NPS Allelix, and Sanofi Aventis.

## References

[1] Khan AA, Hanley DA, Bilezikian JP (2006). Standards for performing dxa in individuals with secondary causes of osteoporosis. J Clin Densitom.

[2] Shapiro CL, Manola J, Leboff M (2001). Ovarian failure after adjuvant chemotherapy is associated with rapid bone loss in women with early-stage breast cancer. J Clin Oncol.

[3] Garrett TJ, Vahdat LT, Kinne DW (1997). Systemic adjuvant therapy of breast cancer. J Surg Oncol.

[4] Jonat W, Kaufmann M, Sauerbrei W (2002). Goserelin versus cyclophosphamide, methotrexate, and fluorouracil as adjuvant therapy in premenopausal patients with node-positive breast cancer: The Zoladex Early Breast Cancer Research Association Study. J Clin Oncol.

[5] Fogelman I, Blake GM, Blamey R (2003). Bone mineral density in premenopausal women treated for node-positive early breast cancer with 2 years of goserelin or 6 months of cyclophosphamide, methotrexate and 5-fluorouracil (cmf). Osteoporos Int.

[6] McCloskey E (2006). Effects of third-generation aromatase inhibitors on bone. Eur J Cancer.

[7] McCloskey E, Hannon R, Lakner G, Clack G, Miyamoto A, Eastell R (2006). The letrozole (l), exemestane (e) and anastrozole (a) pharmacodynamics (leap) trial: a direct comparison to bone biochemical measurements between aromatase inhibitors (ais) in healthy postmenopausal women [abstract 555]. Proc Am Soc Clin Oncol.

[8] Eastell R, Hannon RA, Cuzick J, Dowsett M, Clack G, Adams JE, on behalf of the atac Trialists’ Group (2006). Effect of an aromatase inhibitor on bmd and bone turnover markers: 2-year results of the Anastrozole, Tamoxifen, Alone or in Combination (atac) Trial (18233230). J Bone Miner Res.

[9] Geisler J, Lønning PE, Krag LE (2006). Changes in bone and lipid metabolism in postmenopausal women with early breast cancer after terminating 2-year treatment with exemestane: a randomized placebo-controlled study. Eur J Cancer.

[10] Love RR, Mazess RB, Barden HS (1992). Effects of tamoxifen on bone mineral density in postmenopausal women with breast cancer. N Engl J Med.

[11] Powles TJ, Hickish T, Kanis JA, Tidy A, Ashley S (1996). Effect of tamoxifen on bone mineral density measured by dual-energy X-ray absorptiometry in healthy premenopausal and postmenopausal women. J Clin Oncol.

[12] Fisher B, Costantino JP, Wickerman DL (1998). Tamoxifen for prevention of breast cancer: report of the National Surgical Adjuvant Breast and Bowel Project P-1 Study. J Natl Cancer Inst.

[13] Fisher B, Costantino JP, Wickerham DL (2005). Tamoxifen for the prevention of breast cancer: current status of the National Surgical Adjuvant Breast and Bowel Project P-1 Study. J Natl Cancer Inst.

[14] Baum M, Buzdar A, Cuzick J, on behalf of the atac (Arimidex, Tamoxifen, Alone or in Combination) Trialists’ Group (2003). Anastrozole alone or in combination with tamoxifen versus tamoxifen alone for adjuvant treatment of postmenopausal women with early-stage breast cancer: results of the atac (Arimidex, Tamoxifen Alone or in Combination) trial efficacy and safety update analyses. Cancer.

[15] Thürlimann B, Keshaviah A, Coates AS, on behalf of the Breast International Group (big) 1–98 Collaborative Group (2005). A comparison of letrozole and tamoxifen in postmenopausal women with early breast cancer. N Engl J Med.

[16] Shahinian VB, Kuo YF, Freeman JL, Goodwin JS (2005). Risk of fracture after androgen deprivation for prostate cancer. N Engl J Med.

[17] Coombes RC, Hall E, Gibson LJ, on behalf of the Intergroup Exemestane Study (2004). A randomized trial of exemestane after two to three years of tamoxifen therapy in postmenopausal women with primary breast cancer. N Engl J Med.

[18] Jakesz R, Jonat W, Gnant M, on behalf of the abcsg and the gabg (2005). Switching of postmenopausal women with endocrine-responsive early breast cancer to anastrozole after 2 years’ adjuvant tamoxifen: combined results of abcsg trial 8 and arno 95 trial. Lancet.

[19] Lønning PE, Geisler J, Krag LE (2005). Effects of exemestane administered for 2 years versus placebo on bone mineral density, bone biomarkers, and plasma lipids in patients with surgically resected early breast cancer. J Clin Oncol.

[20] Robson M, Dawson N (1996). How is androgen-dependent metastatic prostate cancer best treated?. Hematol Oncol Clin North Am.

[21] Maillefert JF, Sibilia J, Michel F, Saussine C, Javier RM, Tavernier C (1999). Bone mineral density in men treated with synthetic gonadotropin-releasing hormone agonists for prostatic carcinoma. J Urol.

[22] Daniell HW (1997). Osteoporosis after orchiectomy for prostate cancer. J Urol.

[23] Townsend MF, Sanders WH, Northway RO, Graham SD (1997). Bone fractures associated with luteinizing hormone–releasing hormone agonists used in the treatment of prostate carcinoma. Cancer.

[24] Oefelein MG, Ricchuiti V, Conrad W (2001). Skeletal fracture associated with androgen suppression induced osteoporosis: the clinical incidence and risk factors for patients with prostate cancer. J Urol.

[25] Verhelst J, Denis L, Van Vliet P (1994). Endocrine profiles during administration of the new non-steroidal anti-androgen Casodex in prostate cancer. Clin Endocrinol (Oxf).

[26] Smith MR, Goode M, Zietman AL, McGovern FJ, Lee H, Finkelstein JS (2004). Bicalutamide monotherapy versus leuprolide monotherapy for prostate cancer: effects on bone mineral density and body composition. J Clin Oncol.

[27] Neer RM, Arnaud CD, Zanchetta JR (2001). Effect of parathyroid hormone (1–34) on fractures and bone mineral density in postmenopausal women with osteoporosis. N Engl J Med.

[28] Brown JP, Josse RG, on behalf of the Scientific Advisory Council of the Osteoporosis Society of Canada (2002). 2002 Clinical practice guidelines for the diagnosis and management of osteoporosis in Canada. CMAJ.

[29] Jackson RD, LaCroix AZ, Gass M (2006). Calcium plus vitamin D supplementation and the risk of fractures. N Engl J Med.

[30] Khovidhunkit W, Shoback DM (1999). Clinical effects of raloxifene hydrochloride in women. Ann Intern Med.

[31] Delmas PD, Ensrud KE, Adachi JD (2002). Efficacy of raloxifene on vertebral fracture risk reduction in postmenopausal women with osteoporosis: four-year results from a randomized clinical trial. J Clin Endocrinol Metab.

[32] United States, National Institutes of Health, National Cancer Institute (nci) Study of Tamoxifen and Raloxifene (star) Trial [Web page].

[33] Reszka AA, Rodan GA (2003). Bisphosphonate mechanism of action. Curr Rheumatol Rep.

[34] Meunier PJ, Arlot M, Chavassieux P, Yates AJ (1999). The effects of alendronate on bone turnover and bone quality. Int J Clin Pract Suppl.

[35] Black DM, Cummings SR, Karpf DB (1996). Randomised trial of effect of alendronate on risk of fracture in women with existing vertebral fractures. Fracture Intervention Trial Research Group. Lancet.

[36] Cummings SR, Black DM, Thompson DE (1998). Effect of alendronate on risk of fracture in women with low bone density but without vertebral fractures: results from the Fracture Intervention Trial. JAMA.

[37] Ravn P, Weiss SR, Rodriguez–Portales JA (2000). Alendronate in early postmenopausal women: effects on bone mass during long-term treatment and after withdrawal. Alendronate Osteoporosis Prevention Study Group. J Clin Endocrinol Metab.

[38] Bone HG, Hosking D, Devogelaer JP (2004). Ten years’ experience with alendronate for osteoporosis in postmenopausal women. N Engl J Med.

[39] Hosking D, Chilvers CE, Christiansen C (1998). Prevention of bone loss with alendronate in postmenopausal women under 60 years of age. Early Postmenopausal Intervention Cohort Study Group. N Engl J Med.

[40] Surrey ES, Voigt B, Fournet N, Judd HL (1995). Prolonged gonadotropin-releasing hormone agonist treatment of symptomatic endometriosis: the role of cyclic sodium etidronate and low-dose norethindrone “add-back” therapy. Fertil Steril.

[41] Lindsay R, Adachi JD, Barton IP, Manhart MD (2003). Fracture risk reduction due to antiresorptive treatment is independent of the magnitude of bmd improvement. Arthritis Rheum.

[42] Harris ST, Watts NB, Genant HK (1999). Effects of risedronate treatment on vertebral and nonvertebral fractures in women with postmenopausal osteoporosis: a randomized controlled trial. Vertebral Efficacy With Risedronate Therapy (vert) Study Group. JAMA.

[43] Reginster J, Minne HW, Sorensen OH (2000). Randomized trial of the effects of risedronate on vertebral fractures in women with established postmenopausal osteoporosis. Vertebral Efficacy with Risedronate Therapy (vert) Study Group. Osteoporos Int.

[44] Roux C, Seeman E, Eastell R (2004). Efficacy of risedronate on clinical vertebral fractures within 6 months. Curr Med Res Opin.

[45] Mellström DD, Sörensen OH, Goemaere S, Roux C, Johnson TD, Chines AA (2004). Seven years of treatment with risedronate in women with postmenopausal osteoporosis. Calcif Tissue Int.

[46] McClung MR, Geusens P, Miller PD (2001). Effect of risedronate on the risk of hip fracture in elderly women. Hip Intervention Program Study Group. N Engl J Med.

[47] Mortensen L, Charles P, Bekker PJ, Digennaro J, Johnston CC (1998). Risedronate increases bone mass in an early postmenopausal population: two years of treatment plus one year of follow-up. J Clin Endocrinol Metab.

[48] Dufresne TE, Chmielewski PA, Manhart MD, Johnson TD, Borah B (2003). Risedronate preserves bone architecture in early postmenopausal women in 1 year as measured by three-dimensional microcomputed tomography. Calcif Tissue Int.

[49] Van Poznak C, Hannon RA, Clack G (2007). The sabre (Study of Anastrozole with the Bisphosphonate Risedronate) study: the effects of risedronate on bmd and bone metabolism in postmenopausal women using anastrozole as adjuvant therapy for hormone receptor-positive early breast cancer—first results [abstract 021S]. Bone.

[50] Russell RG (2007). Bisphosphonates: mode of action and pharmacology. Pediatrics.

[51] Ringe JD (2006). Zoledronic acid in the treatment of Paget’s disease and other benign bone disorders. Expert Rev Endocrinol Metab.

[52] Reid IR, Brown JP, Burckhardt P (2002). Intravenous zoledronic acid in postmenopausal women with low bone mineral density. N Engl J Med.

[53] Black DM, Delmas P, Eastell R (2007). Once-yearly zoledronic acid for treatment of postmenopausal osteoporosis. N Engl J Med.

[54] Brufsky A, Harker WG, Beck JT (2007). Zoledronic acid inhibits adjuvant letrozole–induced bone loss in postmenopausal women with early breast cancer. J Clin Oncol.

[55] Watts N, Freedholm D, Daifotis A (1999). The clinical tolerability profile of alendronate. Int J Clin Pract Suppl.

[56] Durie BG, Katz M, Crowley J (2005). Osteonecrosis of the jaw and bisphosphonates. N Engl J Med.

[57] Reid IR, Miller P, Lyles K (2005). Comparison of a single infusion of zoledronic acid with risedronate for Paget’s disease. N Engl J Med.

[58] Hoff AO, Toth B, Altundag K (2005). Osteonecrosis of the jaw in patients receiving intravenous bisphosphonate therapy [abstract 1218]. J Bone Min Res.

[59] Manolagas SC (2000). Birth and death of bone cells: basic regulatory mechanisms and implications for the pathogenesis and treatment of osteoporosis. Endocr Rev.

[60] Lindsay R, Cosman F, Lobo RA (1999). Addition of alendronate to ongoing hormone replacement therapy in the treatment of osteoporosis: a randomized, controlled clinical trial. J Clin Endocrinol Metab.

[61] Cauley JA, Robbins J, Chen Z, on behalf of the Women’s Health Initiative Investigators (2003). Effects of estrogen plus progestin on risk of fracture and bone mineral density: the Women’s Health Initiative randomized trial. JAMA.

[62] Anderson GL, Limacher M, Assaf AR, on behalf of the Women’s Health Initiative Steering Committee (2004). Effects of conjugated equine estrogen in postmenopausal women with hysterectomy: the Women’s Health Initiative randomized controlled trial. JAMA.

[63] Lindsay R, Gallagher JC, Kleerekoper M, Pickar JH (2002). Effect of lower doses of conjugated equine estrogens with and without medroxyprogesterone acetate on bone in early postmenopausal women. JAMA.

[64] Rosen CJ, Chesnut CH, Mallinak NJ (1997). The predictive value of biochemical markers of bone turnover for bone mineral density in early postmenopausal women treated with hormone replacement or calcium supplementation. J Clin Endocrinol Metab.

[65] Rossouw JE, Anderson GL, Prentice RL (2002). (Writing Group for the Women’s Health Initiative Investigators). Risks and benefits of estrogen plus progestin in healthy postmenopausal women: principal results from the Women’s Health Initiative randomized controlled trial. Writing Group for the Women’s Health Initiative Investigators. JAMA.

[66] United States, Food and Drug Administration (fda) fda updates hormone therapy information for postmenopausal women [press release]. FDA News.

[67] McClung MR, Leweicki EM, Cohen SB (2006). Denosumab in postmenopausal women with low bone mineral density. N Engl J Med.

[68] Body JJ, Facon T, Coleman RE (2006). A study of the biological receptor activator of nuclear factor-κB ligand inhibitor, denosumab, in patients with multiple myeloma or bone metastases from breast cancer. Clin Cancer Res.

[69] Rubin MR, Cosman F, Lindsay R, Bilezikian JP (2002). The anabolic effects of parathyroid hormone. Osteoporos Int.

[70] Jilka RL, Weinstein RS, Bellido T, Roberson P, Parfitt AM, Manolagas SC (1999). Increased bone formation by prevention of osteoblast apoptosis with parathyroid hormone. J Clin Invest.

[71] Seeman E (2003). Reduced bone formation and increased bone resorption: rational targets for the treatment of osteoporosis. Osteoporos Int.

[72] Ettinger M, Greenspan S, Marriott T (2005). pth (1–84) prevents first vertebral fracture in postmenopausal women with osteoporosis: the top study [abstract]. Salt Lake City, UT: nps Pharmaceuticals; December 2004, as cited in Shrader SP, Ragucci KR. Parathyroid hormone (1–84) and treatment of osteoporosis. Ann Pharmacother.

[73] Lindsay R, Nieves J, Formica C (1997). Randomized controlled study of effect of parathyroid hormone on vertebral-bone mass and fracture incidence among postmenopausal women on estrogen with osteoporosis. Lancet.

[74] Cosman F, Nieves J, Woelfert L (2001). Parathyroid hormone added to established hormone therapy: effects on vertebral fracture and maintenance of bone mass after parathyroid hormone withdrawal. J Bone Miner Res.

[75] Black DM, Greenspan SL, Ensrud KE, on behalf of the path Study Investigators (2003). The effects of parathyroid hormone and alendronate alone or in combination in postmenopausal osteoporosis. N Engl J Med.

[76] Tashjian AH, Chabner BA (2002). Commentary on clinical safety of recombinant human parathyroid hormone 1–34 in the treatment of osteoporosis in men and postmenopausal women. J Bone Miner Res.

[77] Ma YL, Bryant HU, Zeng Q (2003). New bone formation with teriparatide [human parathyroid hormone-(1–34)] is not retarded by long-term pretreatment with alendronate, estrogen, or raloxifene in ovariectomized rats. Endocrinology.

[78] Marie PJ, Ammann P, Boivin G, Rey C (2001). Mechanisms of action and therapeutic potential of strontium in bone. Calcif Tissue Int.

[79] Boivin G, Deloffre P, Perrat B (1996). Strontium distribution and interactions with bone mineral in monkey iliac bone after strontium salt (S 12911) administration. J Bone Miner Res.

[80] Marie PJ, Neve J (1996). Effects of strontium on bone formation and bone cells. Therapeutic Use of Trace Elements.

[81] Reginster JY, Deroisy R, Dougados M, Jupsin I, Colette J, Roux C (2002). Prevention of early postmenopausal bone loss by strontium ranelate: the randomized, two-year, double-masked, dose-ranging, placebo-controlled prevos trial. Osteoporos Int.

[82] Reginster JY, Meunier PJ (2003). Strontium ranelate phase 2 dose-ranging studies: prevos and stratos studies. Osteoporos Int.

[83] Meunier PJ, Slosman DO, Delmas PD (2002). Strontium ranelate: dose-dependent effects in established postmenopausal vertebral osteoporosis—a 2-year randomized placebo controlled trial. J Clin Endocrinol Metab.

[84] Marie PJ, Hott M, Modrowski D (1993). An uncoupling agent containing strontium prevents bone loss by depressing bone resorption and maintaining bone formation in estrogen-deficient rats. J Bone Miner Res.

[85] Meunier PJ, Roux C, Seeman E (2004). The effects of strontium ranelate on the risk of vertebral fracture in women with postmenopausal osteoporosis. N Engl J Med.

[86] Reginster JY, Seeman E, De Vernejoul MC (2005). Strontium ranelate reduces the risk of nonvertebral fractures in postmenopausal women with osteoporosis: Treatment of Peripheral Osteoporosis (tropos) study. J Clin Endocrinol Metab.

[87] Body JJ, Bergmann S, Boonen S (2007). Management of cancer treatment–induced bone loss in early breast and prostate cancer—a consensus paper of the Belgian Bone Club. Osteoporos Int.

